# Human microbiome transfer in the built environment differs based on occupants, objects, and buildings

**DOI:** 10.1038/s41598-023-33719-6

**Published:** 2023-04-20

**Authors:** Andrew J. Hoisington, Christopher E. Stamper, Katherine L. Bates, Maggie A. Stanislawski, Michael C. Flux, Teodor T. Postolache, Christopher A. Lowry, Lisa A. Brenner

**Affiliations:** 1 Veterans Health Administration, Rocky Mountain Mental Illness Research Education and Clinical Center (MIRECC), Rocky Mountain Regional Veterans Affairs Medical Center (RMRVAMC), VISN 19, Aurora, CO 80045 USA; 2 Department of Physical Medicine and Rehabilitation, University of Colorado Anschutz Medical Campus, Aurora, CO 80045 USA; 3 Military and Veteran Microbiome Consortium for Research and Education (MVM-CoRE), Aurora, CO 80045 USA; 4 Department of Systems Engineering and Management, US Air Force Institute of Technology, Wright-Patterson Air Force Base, OH 45433 USA; 5 Department of Biology, US Air Force Academy, USAF Academy, CO 80840 USA; 6 Department of Biomedical Informatics, School of Medicine, University of Colorado Anschutz Medical Campus, Aurora, CO 80045 USA; 7 Eastern Colorado Health Care System, Veterans Affairs, Denver, CO 80220 USA; 8 Department of Psychology and Center for Neuroscience, University of Colorado Boulder, Boulder, CO 80309 USA; 9 Department of Psychiatry, University of Maryland School of Medicine, Baltimore, MD 21201 USA; 10 Veterans Health Administration, Mental Illness Research Education and Clinical Center (MIRECC), Baltimore VA Annex, VISN 5, Baltimore, MD 21201 USA; 11 Department of Integrative Physiology and Center for Neuroscience, University of Colorado Boulder, Boulder, CO 80309 USA; 12 Center for Microbial Exploration, University of Colorado Boulder, Boulder, CO 80309 USA; 13 Departments of Psychiatry and Neurology, University of Colorado Anschutz Medical Campus, Aurora, CO 80045 USA

**Keywords:** Environmental microbiology, Engineering

## Abstract

Compared to microbiomes on other skin sites, the bacterial microbiome of the human hand has been found to have greater variability across time. To increase understanding regarding the longitudinal transfer of the hand microbiome to objects in the built environment, and vice versa, 22 participants provided skin microbiome samples from their dominant hands, as well as from frequently and infrequently touched objects in their office environments. Additional longitudinal samples from home environments were obtained from a subset of 11 participants. We observed stability of the microbiomes of both the hand and built environments within the office and home settings; however, differences in the microbial communities were detected across the two built environments. Occupants’ frequency of touching an object correlated to that object having a higher relative abundance of human microbes, yet the percent of shared microbes was variable by participants. Finally, objects that were horizontal surfaces in the built environment had higher microbial diversity as compared to objects and the occupants’ hands. This study adds to the existing knowledge of microbiomes of the built environment, enables more detailed studies of indoor microbial transfer, and contributes to future models and building interventions to reduce negative outcomes and improve health and well-being.

## Introduction

The Human Microbiome Project was a foundational study on the connection between humans and their microbes and included two skin sampling sites, the antecubital fossa and retroarticular crease (both right and left sides)^1^. Grice et al. later expanded knowledge regarding the skin microbiota (sampling 20 skin sites, including hands) and observed temporal and interpersonal variability^2,3^. In a comprehensive review of the bacterial skin microbiota, Byrd et al.^4^ noted that microbes on the skin are dependent on the physiology of the skin, notably temperature and potential of hydrogen (pH). For example, the temperature near skin folds (e.g., groin, armpit) can be 37 °C while the temperature at fingertips may be closer to 30 °C, altering microbes that would thrive at the different sites^5^.

Although generally the skin bacterial microbiota has been found to be relatively stable^6^, the hand microbiota has been noted to have greater temporal variability when compared to microbiota from other skin sites^7^. In a review of 18 research articles on the hand microbiota, Edmond–Wilson^7^ noted that the hand microbiota is in a state of constant flux, and influenced by individuals’ activities (e.g., frequency of hand washing, use of oral antibiotics). The human hand contains three times more bacterial phylotypes compared to the forearm or elbow. Caporaso et al.^8^ provided an early and in-depth study of two participants sampling the hand daily for over 6 months. They observed that microbes on the hand can be variable even between days, but within-subject variability for hands remained less than between-subject variability. Additional observations included the presence of pathogenic bacteria on the hands, as well as gender differences in terms of the hand microbiota. As skin is the largest organ of the human body, and our hands have the most frequent direct physical contact with objects in the environment, the hand microbiota is a logical focus of research regarding the microbiome of the built environment.

The bacterial microbiome of the built environment includes microbes that are found in buildings, means of transportation, and human-made physical surrounds. Researchers over the past decade have observed that the built environment harbors unique microbial communities that are influenced by building conditions (e.g., air flow, windows, doors)^9,10^, occupant behavior (e.g. cleaning, activities, number of people in space)^11^, type of facility (e.g., office, residential, commercial)^12^, and geographic location^13^. Microbes in the built environment tend to be a mixture of microbes found in the outdoor environment and on human skin, with additional contributions from the gut and oral cavity^14,15^. Although research regarding the impact of the microbiome of the built environment remains relatively nascent, findings suggest that the microbiome of the built environment can influence occupant health-related outcomes including those associated with physical (e.g., allergic disease, asthma^16^) and mental health^17^.

The purpose of this study was to increase understanding regarding the longitudinal transfer of the human hand bacterial microbiota to objects in two different built environments, an office setting and in homes. Specifically, analyses were focused on objects in frequent (e.g., computer mouse, computer keyboard) and infrequent (e.g., desk, floors, countertops) contact with the human hand. We hypothesized that objects that were more frequently touched by occupants would have increased microbial sharing. This study is one of only a few longitudinal studies that has investigated the transfer of microbes from occupants to objects in the built environment, and the first to have investigated transfer across two different built environments.

## Results

**Figure 1:**
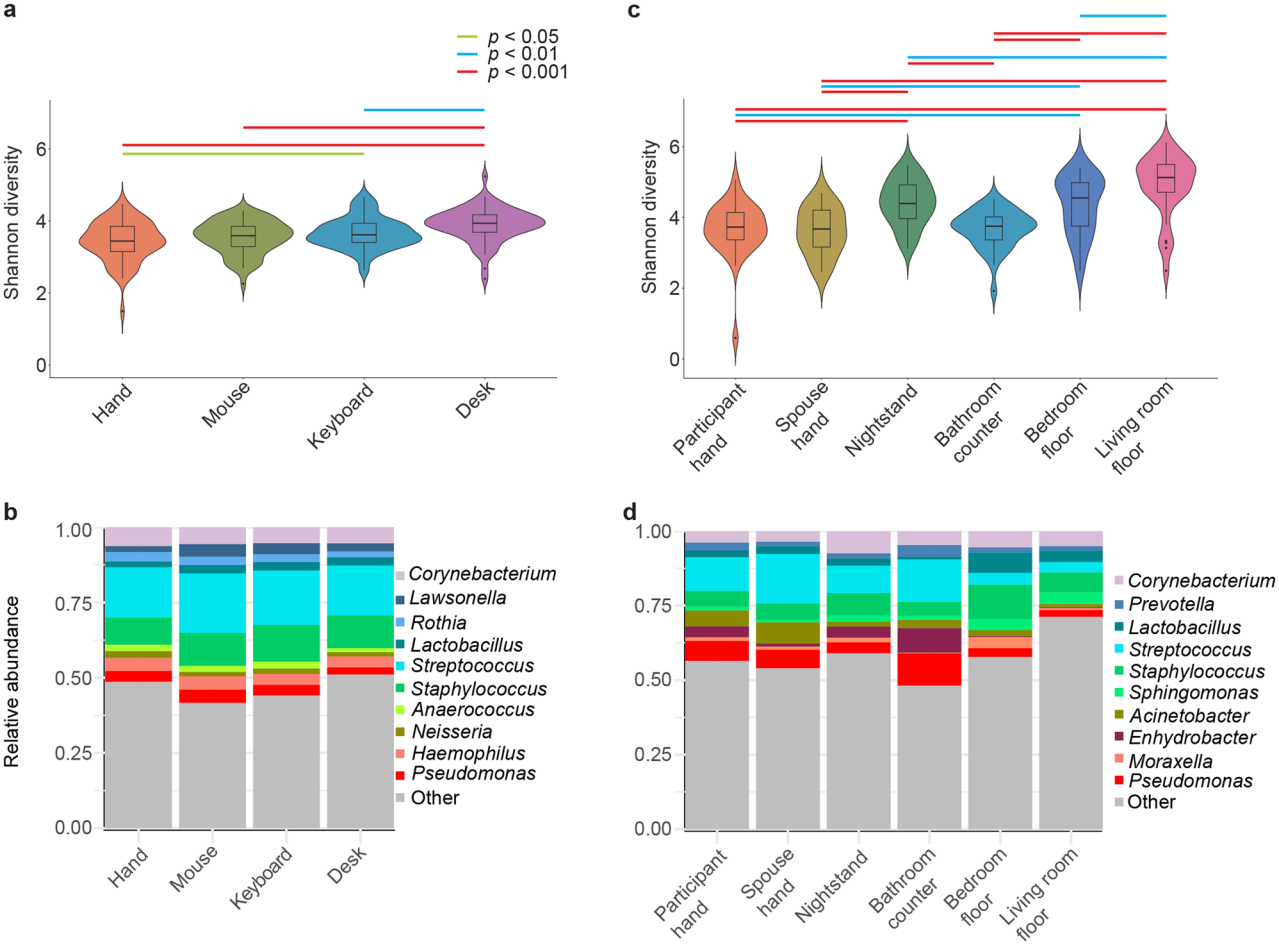
Alpha diversity and top taxa: (**a**) violin plot showing the Shannon diversity metric for samples in the office environment; (**b**) taxonomic summary of observed relative abundances of the top 10 most abundant taxa within each sample type at the genus level for the office environment; (**c**) violin plot showing the Shannon diversity metric for samples in the home environment; and (**d**) taxonomic summary of observed relative abundances of the top 10 most abundant taxa within each sample type at the genus level for the home environment.

Office environment samples were analyzed for alpha diversity, beta diversity, and relative abundance of different taxa. Alpha diversity was the highest in the desk samples compared to all other sample types (desk median Shannon diversity = 3.96). Participants’ hand alpha diversity was similar to the computer mouse and significantly different from the keyboard (Wilcoxon Signed Rank Sum, *p*_FDR-adjusted_ < 0.05) and desk (Wilcoxon Signed Rank Sum, *p*_FDR-adjusted_ < 0.001) (Fig. 1a). Microbial communities collected from participants’ hands, measured by weighted UniFrac distance, were most comparable to the computer mouse and keyboard (Supplementary Fig. 1a). The median weighted UniFrac distances between the hand-mouse and hand-desk were significantly different (Wilcoxon Signed Rank Sum, *p*_FDR-adjusted_ < 0.001). The genera with the highest relative abundances in the office have been reported by other studies as skin-associated bacteria^3,7^. The three most abundant genera were the same for each sample type: *Streptococcus*, *Staphylococcus* and *Pseudomonas* (see Fig. 1b).

Home environmental samples were analyzed using the same outcomes as the office environment. Alpha diversity and the taxonomic profiles were driven by sample type, that is skin, horizontal surface, or floor (Supplementary Fig. 1b). Floor samples showed the highest alpha diversity, led by the living room floor (median Shannon diversity = 5.6) and bedroom floor (median Shannon diversity = 5.1). The alpha diversity of the participants’ hands was not different from that of the partner’s hand or bathroom counter, but was different from the nightstand (Wilcoxon Signed Rank Sum, *p*_FDR-adjusted_ < 0.001), bedroom floor (Wilcoxon Signed Rank Sum, *p*_FDR-adjusted_ < 0.01), and living room floor (Wilcoxon Signed Rank Sum, *p*_FDR-adjusted_ < 0.05) (Fig. 1c). Beta diversity of the participants’ hands was most like the partner’s hand and significantly different from living room floor (weighted UniFrac; PERMANOVA, *p* < 0.05; Supplementary Fig. 1b). The top ten genera in the home were driven by sample type with floor, raised surfaces, and skin samples showing similarity to themselves, but dissimilarity to other sample types (Fig. 1d).

### Microbial communities of the built environment and human hands were stable

**Figure 2:**
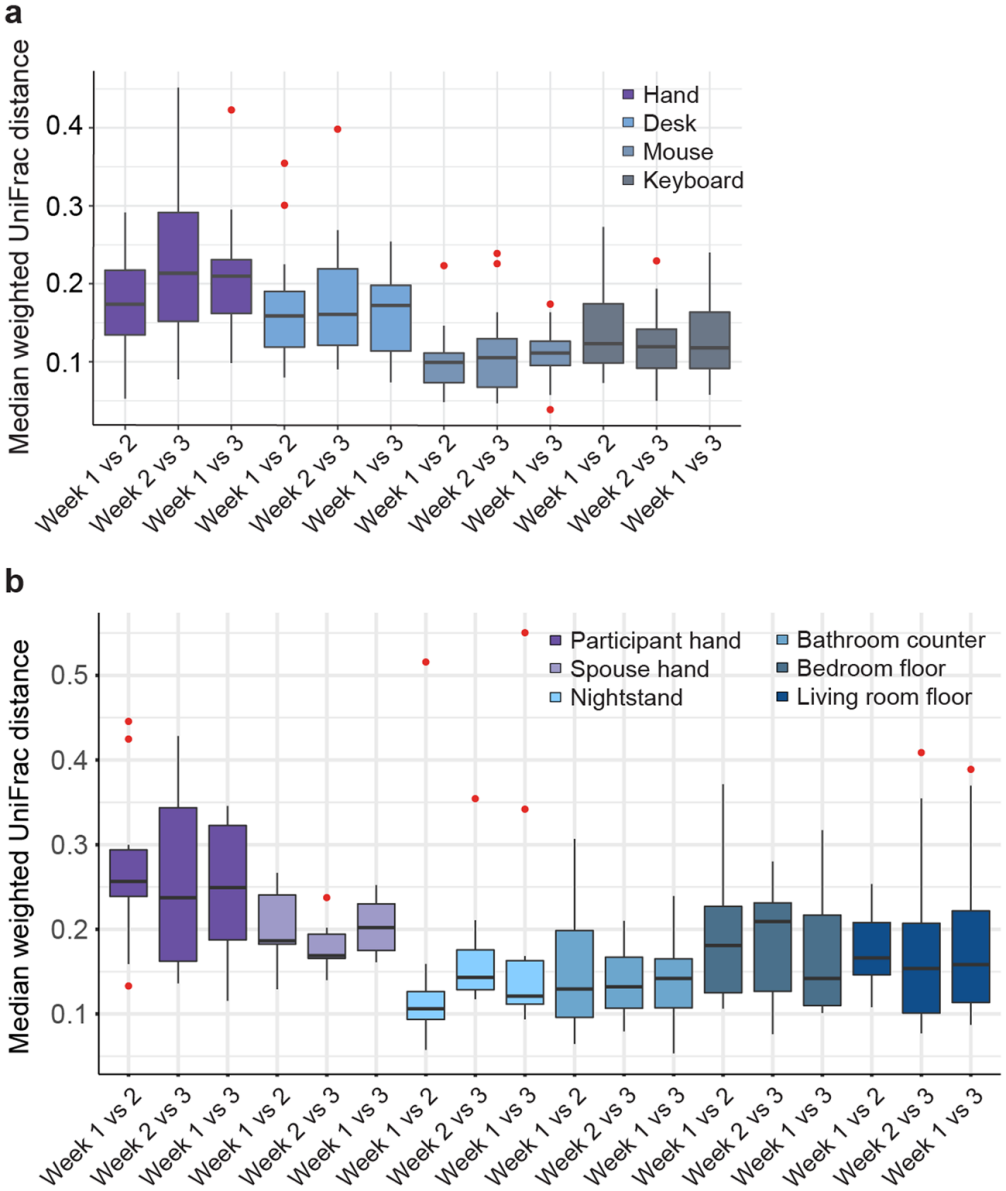
Longitudinal stability of microbial communities (weighted UniFrac) within each participant colored by sample type in the office and home environments: (**a**) boxplot representing median weighted UniFrac distances within each participant colored by sample type between the weeks of sampling for the office environment. Sample sizes can be found in Supplementary Table 1; (**b**) boxplot representing median weighted UniFrac distances within each participant colored by sample type between pairs of the weeks of sampling for the home environment. Sample sizes can be found in Supplementary Table 2.

Alpha and beta diversity were longitudinally stable for the 3-week sampling effort within each sample type for both the office and home. The 3 weeks of sampling for the office and home each had similar Shannon diversity across time (all linear mixed model (LMM) *p* > 0.05; Supplementary Fig. 2a,b). Likewise, analysis by LMM determined that beta diversity, within each participant among sampling weeks, was stable in both the office and home (all LMM *p* > 0.05; Fig. 2a,b). In the office, the most stable microbial communities were on the computer mouse and least stable on participants’ hand. In the home, the most stable microbial communities were on the nightstand and bathroom counter, and least stable on the hands (both partner and participant).

### Direct and frequent contact increased microbial sharing between the hand and built environment

**Figure 3:**
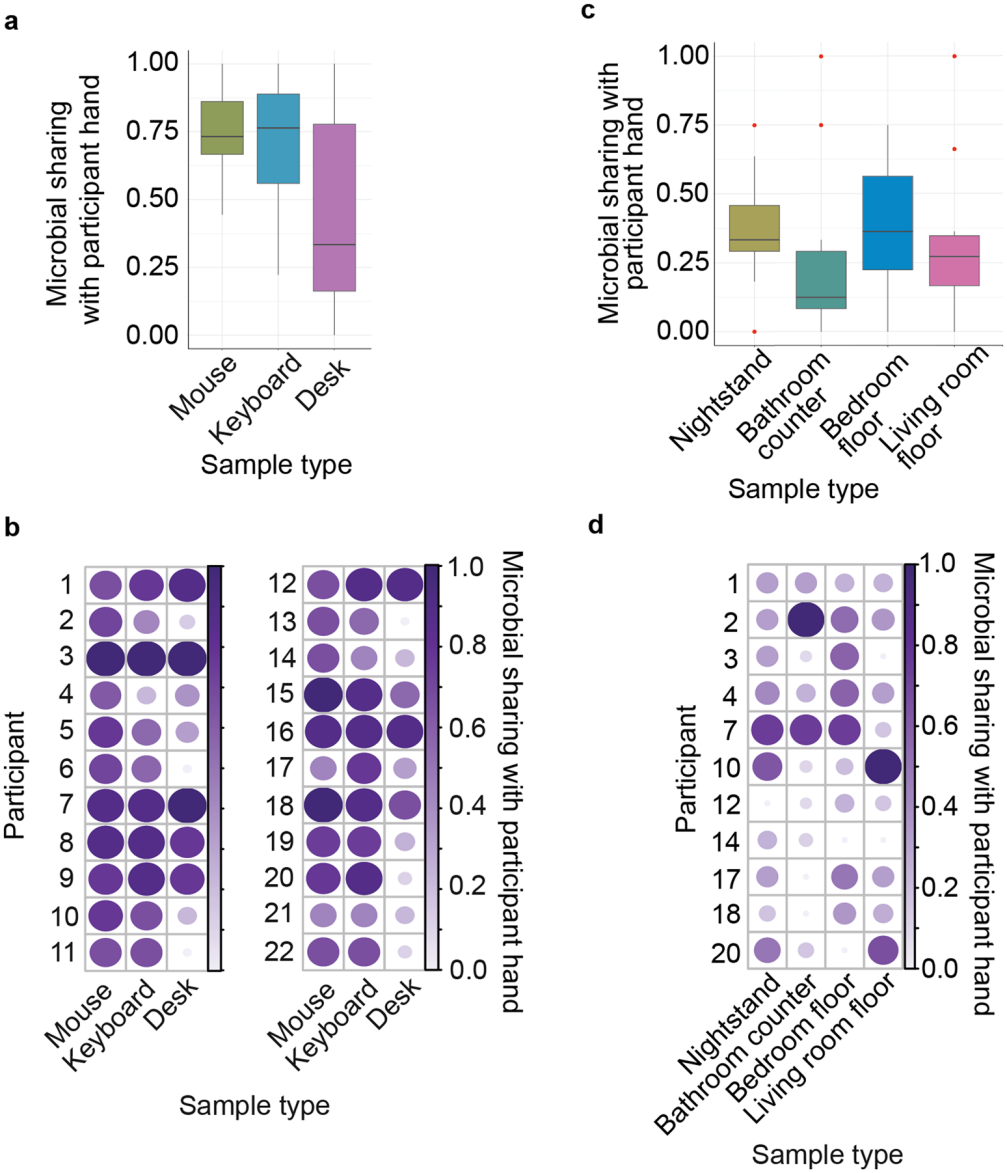
Microbial sharing in the *built* environment: (**a**) boxplot of microbial sharing with the participant hand by sample type in office. Sample sizes for each sample type were mouse (*n* = 65); keyboard (*n* = 64); desk (*n* = 63); (**b**) circle plot representing the microbial sharing by participant stratified by sample type in office. Lager circles and darker purple represent higher proportions shared communities. Sample sizes for each participant in office can be found in Supplementary Table 3; (**c**) boxplot representing the microbial sharing with the participant hand by sample type in home. Sample sizes for each sample type were nightstand (*n* = 31); bathroom (*n* = 33); bedroom floor (*n* = 31); living room floor (*n* = 31); (**d**) circle plot representing microbial sharing by participant stratified by sample type. Larger circles and darker purple represent higher proportions of shared communities. Sample sizes for each participant in home can be found in Supplementary Table 4. Partner hand samples were removed from these figures because not all participants had a partner.

One method used to analyze microbial transfer between the hand and the built environment was random forest modelling. Samples in direct and frequent contact with the participant showed higher degrees of microbial transfer. Specifically, the hand, mouse, and keyboard each displayed the highest degree of microbial sharing (Fig. 3a). In contrast, the desk surface showed far less microbial sharing with the hand microbiome. Participants displayed high individual variability in the levels of microbial transfer to the built environment (Fig. 3b; Supplementary Fig. 3a). Participant hand microbiome that were less diverse across the study period had increased prediction accuracy (weighted UniFrac distance; Pearson correlation, *p* = 0.2; Supplementary Fig. 4).

There was less microbial sharing between the participant and the built environment in the home relative to the office. The participants’ hands shared the most with the nightstand and least with the bathroom counter (Fig. 3c). However, like the office, there was a high level of interindividual variability in the level of microbial transfer (Fig. 3d; Supplementary Fig. 3b). Participant hand microbiome that were less diverse across the study period had decreased prediction accuracy which was the opposite of the trend observed in the office (weighted UniFrac distance; Pearson correlation, *p* = 0.03); Supplementary Fig. 4).

### Similar sample types shared more OTUs

**Figure 4:**
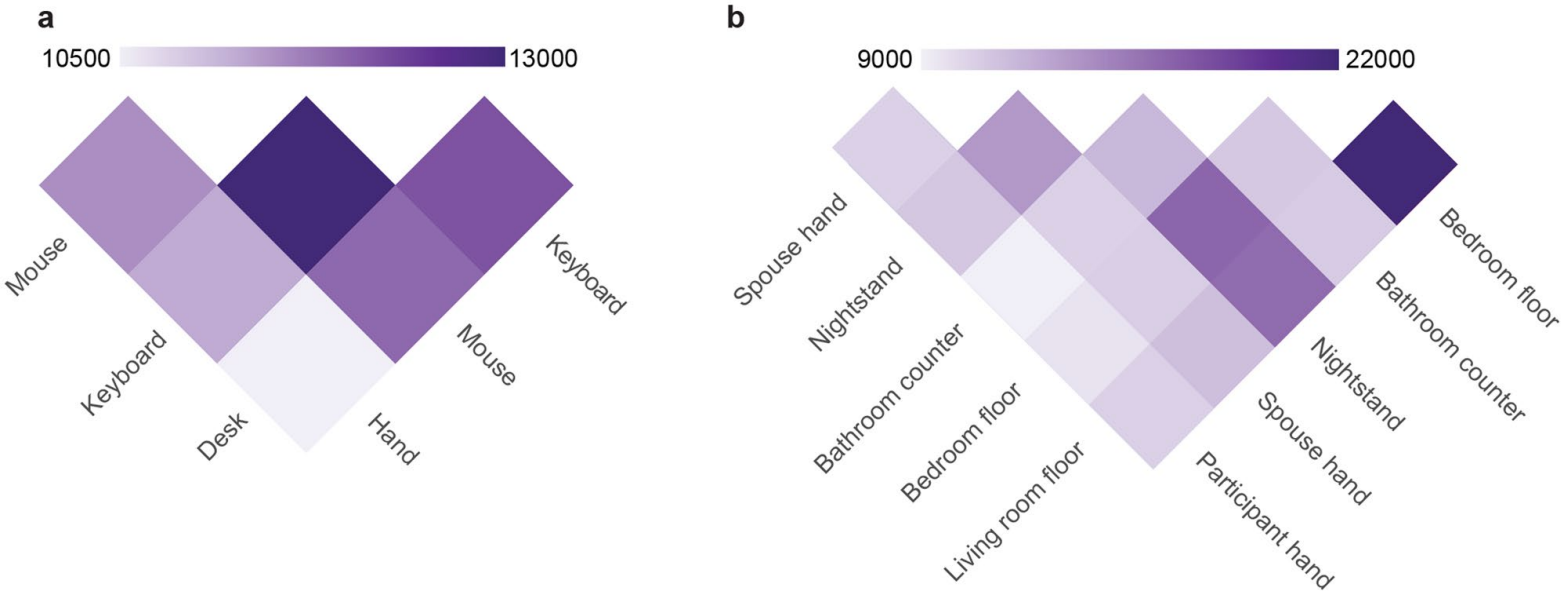
Shared OTUs by sample type: (**a**) heat map representation of the summed shared OTUs between each sample type for the office environment; (**b**) heat map representation of the summed shared OTUs between each sample type for the home environment.

Sample types that were similar shared more operational taxonomic units (OTUs). In the office, all the items on the desk shared more OTUs with each other than with the hand (Fig. 4a). Specifically, the keyboard and mouse share the most OTUs, while the hand and desk shared the least OTUs. In the home, the bedroom and living room floors shared the most OTUs, while the partner hand and bathroom counter shared the least OTUs (Fig. 4b). Interestingly, the nightstand and floor samples shared many OTUs.

### Limited microbial sharing across office and home environments

A subset of participants (*n* = 11) had both office and home samples. Shannon diversity in the hand samples of these participants was longitudinally stable over the 6 weeks of sampling (LMM, *p* = 0.28) (Supplementary Fig. 5a). However, microbial sharing was low across the office and home environments. This was exemplified by more sharing in samples from the same environment relative to samples across the two environments (Supplementary Fig. 5b,c). The computer mouse shared more microbial material with the hand sample from the office relative to the hand samples across environments. Intriguingly, participants office hand sample shared more material with their partner hand than their own hand sample from the home environment (Supplementary Fig. 5c). Analysis of beta diversity revealed that the microbial communities of the office and home were different (PERMANOVA, *p* < 0.001; Supplementary Fig. 6).

## Discussion

In the present study, microbiome samples collected longitudinally from participants hands were analyzed and compared with microbiome samples recovered from objects in office and residential environments. Key findings from this study are as follows: (1) microbiota stability of both the hand and built environment was observed within the office and home settings; however, this same stability was not observed across the two environments; (2) objects and surfaces in the built environment in more frequent contact with occupants were more microbially similar to the occupants’ hands than those in less frequent contact; (3) the degree of sharing of the microbial community to the built environment was variable across participants; and (4) horizontal surfaces (e.g., desk, nightstand, living room floor) in the built environment had increased microbial diversity as compared to objects or the occupants hands.

Consistent with previous research, microbes from the human hands and built environment surfaces at each location were relatively stable across the 3 weeks of sampling^14,18^. Hand microbial stability was consistently lower than that from sampled objects in the built environment. Interestingly, while our study showed stability in both the office samples and home samples, over the 3-week sampling period, hand microbiota across the two locations were observed to be less stable. In addition, compared to the office environment, the home environment had reduced shared microbial communities between the hand and the built environment. To the best of our knowledge, direct comparisons between office and home environments for the same participants had previously not been conducted. Several factors might influence the lack of hand microbial sharing observed in our study. Compared to a more sterile office environment, the home environment had additional sources of microbes to include: food preparation, storage, and consumption^19^; increased connection to the outdoor environment through the opening of windows, doors, and outdoor air exchange^10^; and multiple human and non-human biological carriers^12^. An influencing factor (gaining increased study at present) on microbial communities could be anthropogenic chemicals that may even be bio-transformed by microbes-especially in residential environments that have water present^20^. Additionally, the reduction in microbial sharing observed in the home compared to the office could be related to the materials we sampled that were primarily surfaces that were not in contact with the hand as much as in the office. Finally, a break of approximately 1 month between the completion of the office study and the start of the home study could have altered the results.

In our study, surfaces and objects in the built environment that were more likely to be touched had more similar microbes to the occupants. Meadow et al.^21^ investigated classroom surfaces and observed similar findings, with more skin associated bacteria on desk and chairs compared to walls and floors. Details on the quantity of microbial transfer that occurs and the influence of surface characteristics on transfer efficiency are still nascent. However, a recent uniquely designed study by King et al.^22^ used a Bayesian approach to estimate *E. coli* transfer during multiple surface contacts by either bare skin or gloved fingers. Their main research findings included: skin was not as efficient as transferring *E. coli* compared to gloved hands, after six touches the transfer of *E. coli* from the finger to the surface reached a plateau where increased touches did not deposit more bacteria, and the average transfer efficiency of *E. coli* was 49% (± 12%) for an ungloved hand. Another study by Wang et al.^23^ observed in the built environment that a single bacterial organisms could be tracked to an occupant, frequent hand washing did not prevent the bacteria of interest from being increasingly prevalent on hands compared to inanimate objects, and objects closer to an occupant had a greater biological signature of the occupant. A broader study of the microbiota could identify both microbes and amounts that are transferred, possibly a product of the surface type. A small study of three participant pairs did observe grouped microbial communities in the pairs (assessed using Jaccard Similarity), that were transferred indirectly when participants touched paper, glass, and cotton substrates^24^.

Transfer of human microbial communities to surfaces in the environment was variable across the study. For example, we predicted the correct desk to hand microbiota with under 50% accuracy for 13 participants and over 75% accuracy for 7 participants (including two at 100%). We did not survey details on the built environment or occupant behavior that might have contributed to the differences. Therefore, it is not possible to ascertain if the increased transfer observed was due to occupant behavior (e.g., touching the desk more often, cleaning less, having fewer visitors) or the hand microbes being more distinct for select individuals. Horizontal surfaces had more variability in the percentage of transfer from the hand microbiota, as clear from data from sampling of the home environment of all horizontal surfaces. Variable accuracy between subject and objects has been noted in other studies^25,26^, potentially confounded by sampling time (not measured in the current study) with diurnal variation in the bacterial skin microbiota^26^.

Historically, the transfer of microbes from occupants has been studied at the level of one or several taxa with the focus on infectious disease transfer. This research has occurred predominately in sensitive locations like hospitals^27,28^ or more recently during the COVID-19 pandemic to identify infection risk from surfaces (though the SARS-CoV-2 virus.is mainly an airborne pathogen with risk of infection through surface transmission estimated at < 10^–6^^29^). Information gleaned from future studies of fomite and human infectious disease interactions is expected to provide valuable foundational knowledge for research regarding the microbiome of the built environment.

Horizontal surfaces in the office and home environment were observed to be both more diverse (higher alpha diversity) and more stable (lower beta diversity across the weeks). These findings are consistent with our previous study on the microbiome of the built environment at the United States Air Force Academy^14^. The additional diversity observed on horizontal surfaces is due to particle deposition, occupant behavior (e.g., cleaning, indoor activities), and potential presence of a domesticated animal. Desquamation is another source of microbes on horizontal surfaces, originating from multiple sources on the human body besides the hand. Therefore, it was unsurprising that the horizontal surfaces had the least percentage of hand microbes in the present study. Even in the office environment that had a desk only used by the one participant, the hand microbes shared was on average under 30% (with least shared OTUs for all sites).

More recently, the concept of using the microbiome of the built environment as a forensic tool has been discussed in multiple research papers. Although not a central aim of this study, one method that was used (random forest), has been also used in previous microbial forensic research endeavors. Our results suggest that forensic insights would be best gleaned from: (1) items that are most frequently contacted by occupants; (2) assessment of human microbiome samples from microbial reservoirs that contact the built environment (e.g., hands); and (3) assessment of offices compared to residences. The best result for microbial forensics in our study was approximately 75% accuracy for the hand and the computer mouse, far exceeding random chance (4.3%), but also considerably less than the gold standard of DNA (99.8% accuracy). Moreover, it is unclear why two individuals had under 50% matching between hand microbiota and the computer mouse while three participants had a 100% match. A deeper understanding on the impacts of external factors on the human and built environment microbiome is warranted. Researchers have suggested that microbiome forensics (at least that based on 16S rRNA gene sequencing) can augment other tools to provide additional information^30^, and can be used to detect meaningful changes to occupants in the built environment (e.g., resulting from exposures to psychosocial stress)^17^, or even provide postmortem information^31^. The field of microbiome forensics is at a very early stage but has significant potential for future exploration and application.

The present study has several strengths and limitations. The longitudinal design enabled analysis of microbiota stability over time; however, measuring beyond 3 weeks we might have resulted in different conclusions. Additionally, the small sample size (22 participants in offices, 11 participants in homes) and only one study site limit the strength of conclusions that would be possible from a larger multi-location study. Indeed, all of our participants were working in the same office building in the same city. Nonetheless, the use of two locations inhabited by the same occupant is unique and provided evidence that the built environment influences the hand microbiome and the detection of the human microbiome on objects and surfaces indoors. Sampling methodology, DNA extraction processes, and primer selection can also influence results of skin studies^32–34^. For example, Mesiel et al^35^ recommended the hypervariable region 2 (V2) of the 16S rRNA gene for sequencing skin microbiota, in part due to its ability better ability to more readily detect *Propionibacterium*. The human hand does not have appreciable amounts of *Propionibacterium*^2^, yet we recognize a different methodology or primer set could alter the results we observed. Finally, sampling occurred prior to the COVID-19 pandemic and future studies could be more impacted by improved hand hygiene, an increased use of hand sanitizers, and occupational changes (e.g. avoidance of individual, fist bumps instead of hand shakes, etc.).

Transfer of microbes between an occupant and the built environment is dependent upon many factors, some that have yet to be uncovered. A fundamental question remains on how different built environments and occupants might change the microbiome of the built environment. With an improved understanding of the basic processes involved, accurate models and building interventions could be developed-reducing negative outcomes (e.g., disease transmission) and, in parallel, improve occupant health and well-being.

## Methods

### Participants and study design

This study included two distinct sample collection periods, one in the office environment and one in a home environment. The office study 22 participants were recruited who each occupied a single-occupant office of approximately the same size located in the same building. On a weekly basis for three consecutive weeks the participants’ dominant palm, entire computer mouse, entire keyboard, and a one square foot area of desk space were swabbed by an investigator. Each participant was the sole occupant for that desk although visitors to the participant were allowed during the study period. Approximately 1 month after the office study sampling was completed, the home samples began for a subset of participants from the office study (*n* = 11). Participants were asked to self-sample their home environment for three consecutive weeks. The participants collected samples from their dominant palm, bedroom nightstand, bathroom counter, bedroom floor, and living room floor. In addition, when present, adult partners of the participant self-sampled their dominant palm (*n* = 6). All sampling was done pre-COVID pandemic with participants working in the office approximately 40 h in a 5-day work week.

Participants were faculty and staff at the Air Force Academy and resided within close proximity to Colorado Springs, Colorado, USA. This study was conducted according to the World Medical Association guidelines in the Declaration of Helsinki that provide ethical principles for medical research involving human subjects^36^, and all procedures involving human participants were approved by the Air Force Academy Institutional Review Board (IRB Protocol: FAC20160003H). Written informed consent was obtained before individuals participated in any study procedures. Additionally, the study was given exempt status from the University of Colorado Boulder Institutional Review Board as data were de-identified and no further contact with participants was necessary in the analysis.

### Sample collection and preparation

BD BBL™ CultureSwab™ EZ II polyurethane sponge swabs (Cat. No. 220145, Becton, Dickinson and Company, Sparks, MD, USA) dipped in sterile (autoclaved) phosphate buffer solution with DNA-free water, were used to swab all surfaces except for the floor samples. Floor samples were obtained with a thimble filter (Cat. No. 8715600 EMSL, Cinnaminson, New Jersey, USA) attached to a vacuum hose. Collection of microbial biomass was obtained by vacuuming a one-square foot section of the floor. All samples from the office study were stored at − 80 °C within an hour of sampling. All samples from the home study were stored at − 20 °C for up to 24 h immediately following sampling, then moved to − 80 °C. All samples were transported by car to the University of Colorado Boulder (~ 90 miles) on dry ice for further processing.

### Molecular processing

DNA was extracted using the PowerSoil DNA extraction kit (Cat No. 12888-100 and 12955-4, MoBio Laboratories, Carlsbad, CA, USA) according to the manufacturer’s instructions. Marker genes in isolated DNA were PCR-amplified using HotStarTaq Master Mix (Cat No. 203433, Qiagen, Valencia, CA, USA) and 515 F (5′-GTGCCAGCMGCCGCGGTAA-3′)/806 R (5′-GGACTACHVGGGTWTCTAAT-3′) primer pair (Integrated DNA Technologies, Coralville, IA, USA) targeting the V4 hypervariable region of the 16S rRNA gene modified with a unique 12-base sequence identifier for each sample and the Illumina adapter, as previously described^37^. The thermal cycling program consisted of an initial step at 94 °C for 3 min followed by 35 cycles (94 °C for 45 s, 55 °C for 1 min, and 72 °C for 1.5 min), and a final extension at 72 °C for 10 min. PCR reactions were run in duplicate and the products from the duplicate reactions were pooled and visualized on an agarose gel to ensure successful amplification. PCR products were cleaned and normalized using a SequalPrep Normalization Kit (Cat. No. A1051001, ThermoFisher, Waltham, MA, USA) following manufacturer’s instructions. The normalized amplicon pool was sequenced on an Illumina MiSeq run using V3 chemistry, 600 cycles, and 2 × 300-bp paired-end sequencing. All sequencing and library preparation were conducted at the University of Colorado Boulder BioFrontiers Next-Gen Sequencing core facility.

### Computational analysis

Raw sequences were trimmed, demultiplexed, merged, quality filtered (maxee value of 1 and singletons removed), and clustered into greater than or equal to 97% similar phylotypes using UPARSE 8^38^. Quality reports from the fastq_eestats2 command were used to determine the fixed length at which the raw sequences were trimmed prior to merging (forward read: 200 nucleotides; reverse read: 150 nucleotides). Taxonomy was assigned using the Silva (version 138) 16S rRNA gene database^39^. For downstream analysis, samples were rarefied to a depth of 1500 and 4130 sequences for the office study and home study, respectively. In analyses where both experiments were examined together, samples were rarefied to a depth of 1500. Quantitative Insights Into Microbial Ecology (QIIME v 1.9.1) was used to generate weighted UniFrac distances to examine the microbial community structure^40,41^. Further computational analysis and figure generation were performed in R (v 3.4.1). Analysis of alpha and beta diversity was performed with the R packages phyloseq (v 1.20.0)^42^ and mctoolsr (0.1.1.1) https://github.com/leffj/mctoolsr. Bacterial community composition differences between: (1) office and home environments, (2) sample types, and (3) participants were tested with permutational analysis of variance (PERMANOVA), performed with the vegan (v 2.4-4) package in R using weighted UniFrac distance matrices^43^. All microbial community measures in the present study utilized weighted UniFrac, accounting for the phylogenetic variance and relative abundance of operational taxonomic units. For each sample type, we used linear mixed modeling as a function of time, accounting for the correlation by participant, to model outcomes of median weighted UniFrac distance and Shannon diversity. Comparisons of Shannon diversity metric by sample type were determined through Wilcoxon Signed Rank-Sum test. Linear mixed model analysis was performed with the nlme (v 3.1-159) package in R. Pearson correlation was performed to assess the association between longitudinal stability and prediction accuracy. False Discovery Rate was used to adjust for multiple comparisons^44^. All statistics were performed in R (v 3.4.1).

The QIIME command shared_phylotypes.py was used to gather the shared OTUs between different sample types that were collapsed using the collapse_samples.py (normalized and in sum mode). In the home study*,* a correction factor was applied to the collapsed samples of the partner’s hand because there were only 6 participants that had partner samples of the 11 total participants.

Random forest analyses were done with the caret (v 6.0-77) and randomForest (4.6-12) packages in R. Random Forest models were generated using the repeated tenfold cross-validation method repeated 3 times with default parameters. Genus level taxa were used to predict participant, excluding low prevalence taxa (taxa that were present in less than 10 samples). Random forests were run on all data for each environment (office and home, Figs. 3a and 4a respectively). Subsequent random forests were run using each sample type to train a model to test on the remaining sample types within each environment (Fig. 3; Supplementary Table 3 for office, Fig. 4; Supplementary Table 4 for home). The iterative modeling approach allowed for the predictive capability of individual sample types to be assessed and to alleviate concerns of “overfitting” that arose with the high accuracy of the random forests that included all sample types. The practice of separating the training and testing data ensured that the models were never exposed to the testing data prior to making predictions.

The datasets for this study can be found in the QIITA under study ID 14787 https://qiita.ucsd.edu/study/description/14787 and EBI-ENA under the accession number PRJEB56766. This manuscript complies with the STORMS reporting checklist, v1.03^45^.

## Supplementary Information

Supplementary Information.

## Data Availability

The datasets for this study can be found in QIITA under study ID 14787 https://qiita.ucsd.edu/study/description/14787 and EBI-ENA under the accession number PRJEB56766.
