# Supplementary material for: Human microbiome transfer in the built environment differs based on occupants, objects, and buildings
Source: Sci Rep. 2023 Apr 20;13:6446. doi: 10.1038/s41598-023-33719-6 (PMC10116103; doi:10.1038/s41598-023-33719-6)
Supplement: Supplementary file 1 — Supplementary Information. [file 41598_2023_33719_MOESM1_ESM.docx]

# Supplementary Material


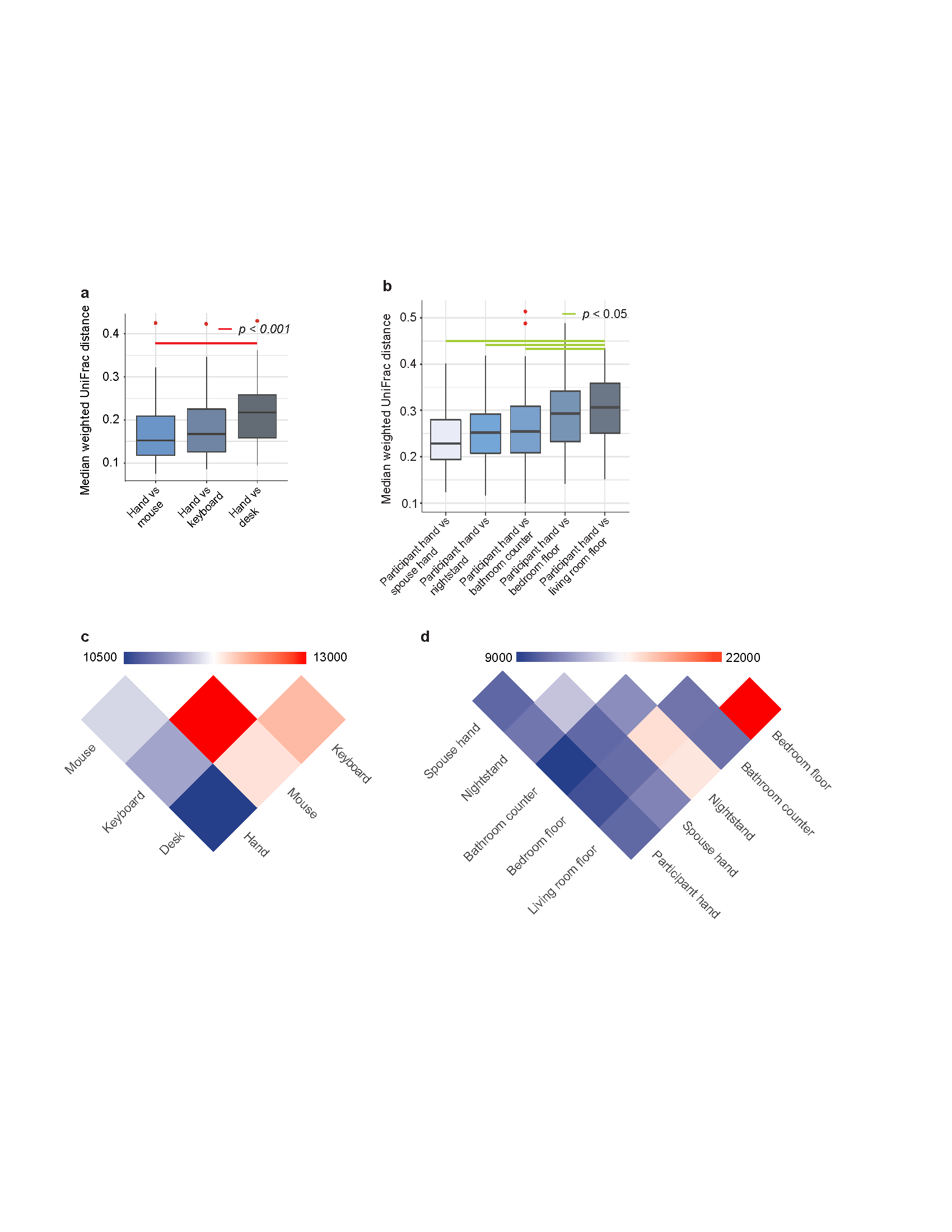


**Supplementary Figure 1**. **Longitudinal stability of weighted UniFrac Distance:** **a.** boxplot representing median weighted UniFrac distances between the hand and all other sample types in the office environment; **b.** boxplot representing median weighted UniFrac distances between the hand and all other sample types in the home environment.


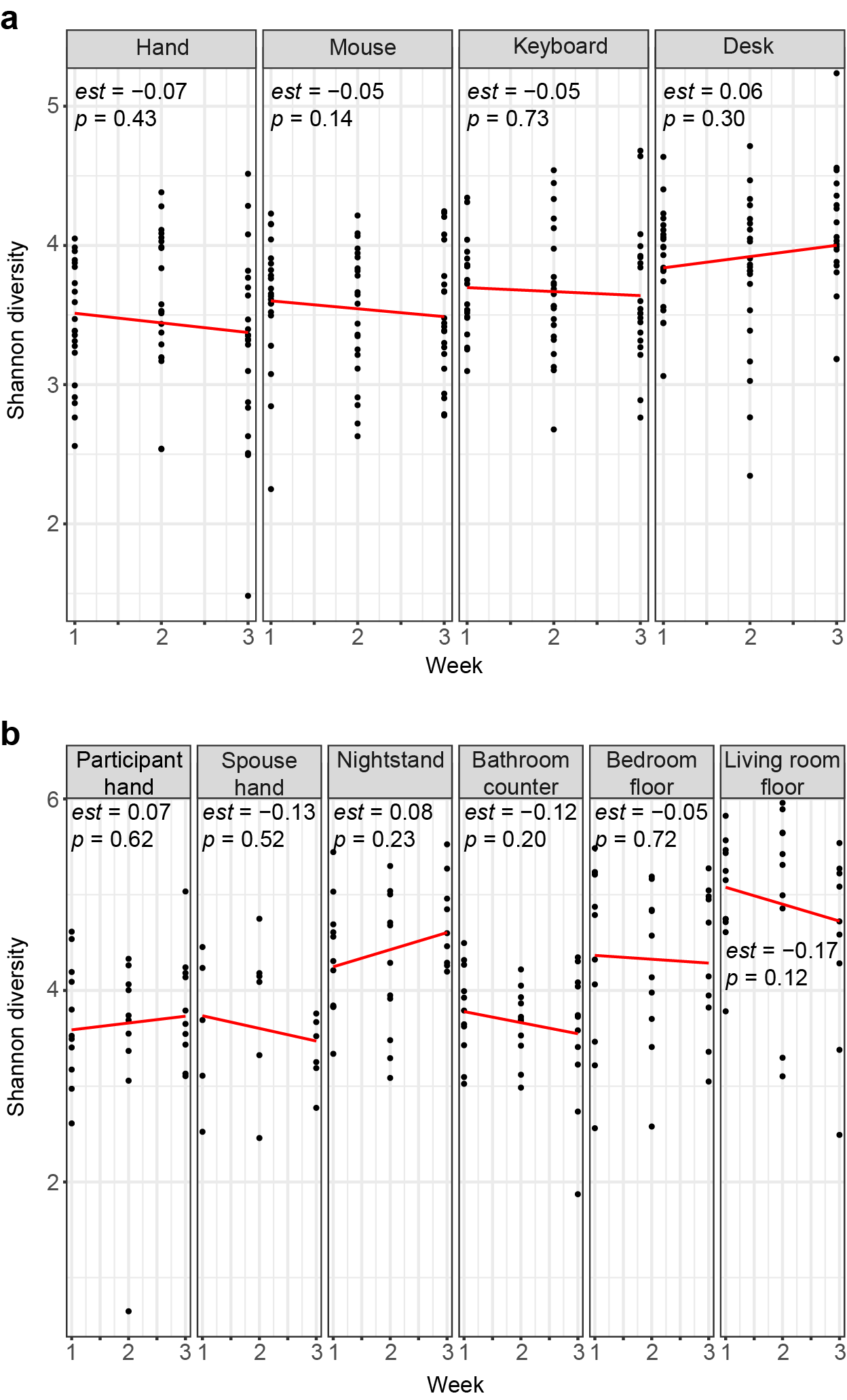


**Supplementary Figure 2. Longitudinal stability of Shannon diversity: a.** scatter plots of Shannon diversity from office; **b**. scatter plots of Shannon diversity from home environments. (red lines = trend line, *estimate* and *p* values from LMM). Sample sizes can be found in *Supplementary Table 1* (office) and *Supplementary Table 2* (home) of the supplemental information.


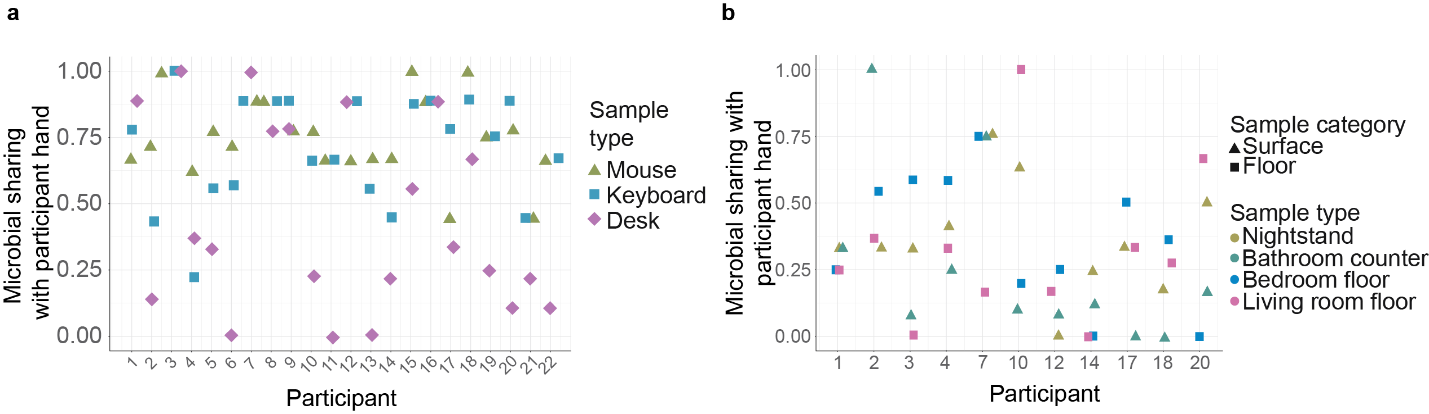


**Supplementary Figure 3**. **Microbial sharing in the built environment:** **a.** Jitter plot representing the microbial sharing by participant stratified by sample type in the office; **b.** Jitter plot representing the microbial sharing by participant stratified by sample type in the home.


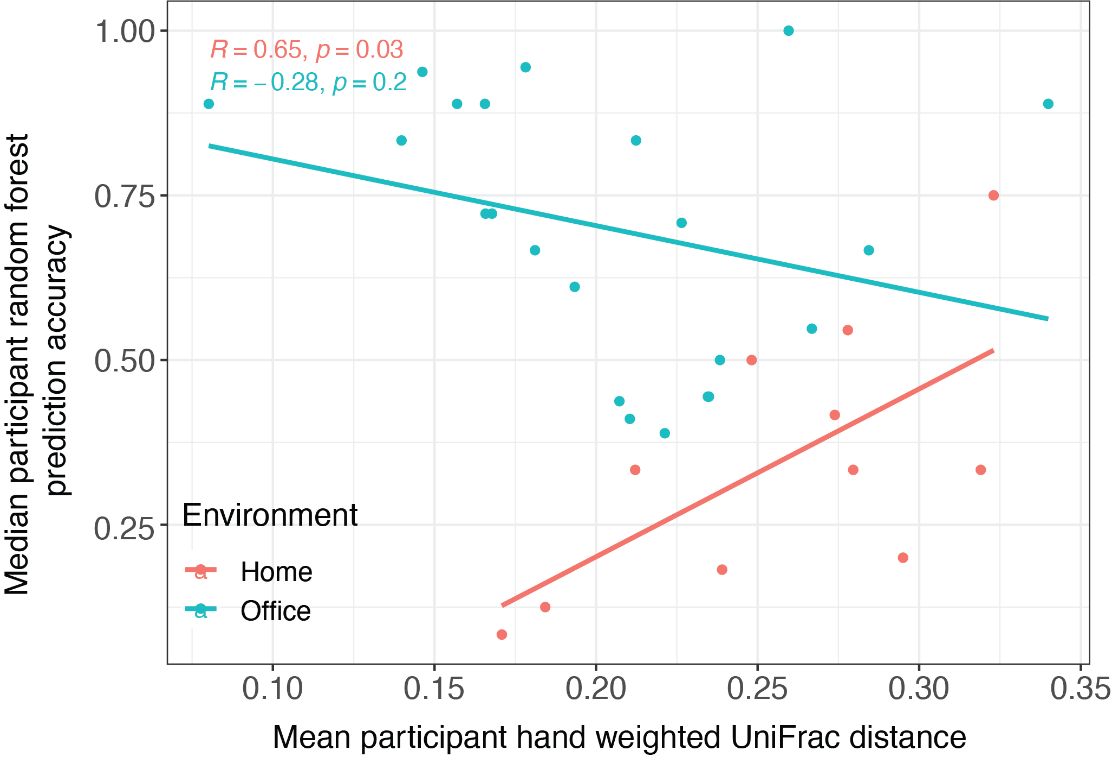


**Supplementary Figure 4. Participant hand longitudinal stability vs random forest prediction accuracy.** Scatter plot of mean weighted UniFrac distances of within-participant hand between weeks vs median prediction accuracy of all surface types for each participant separated by environment. Trend lines and statistics are Pearson correlation coefficient and *p-*value for each environment.


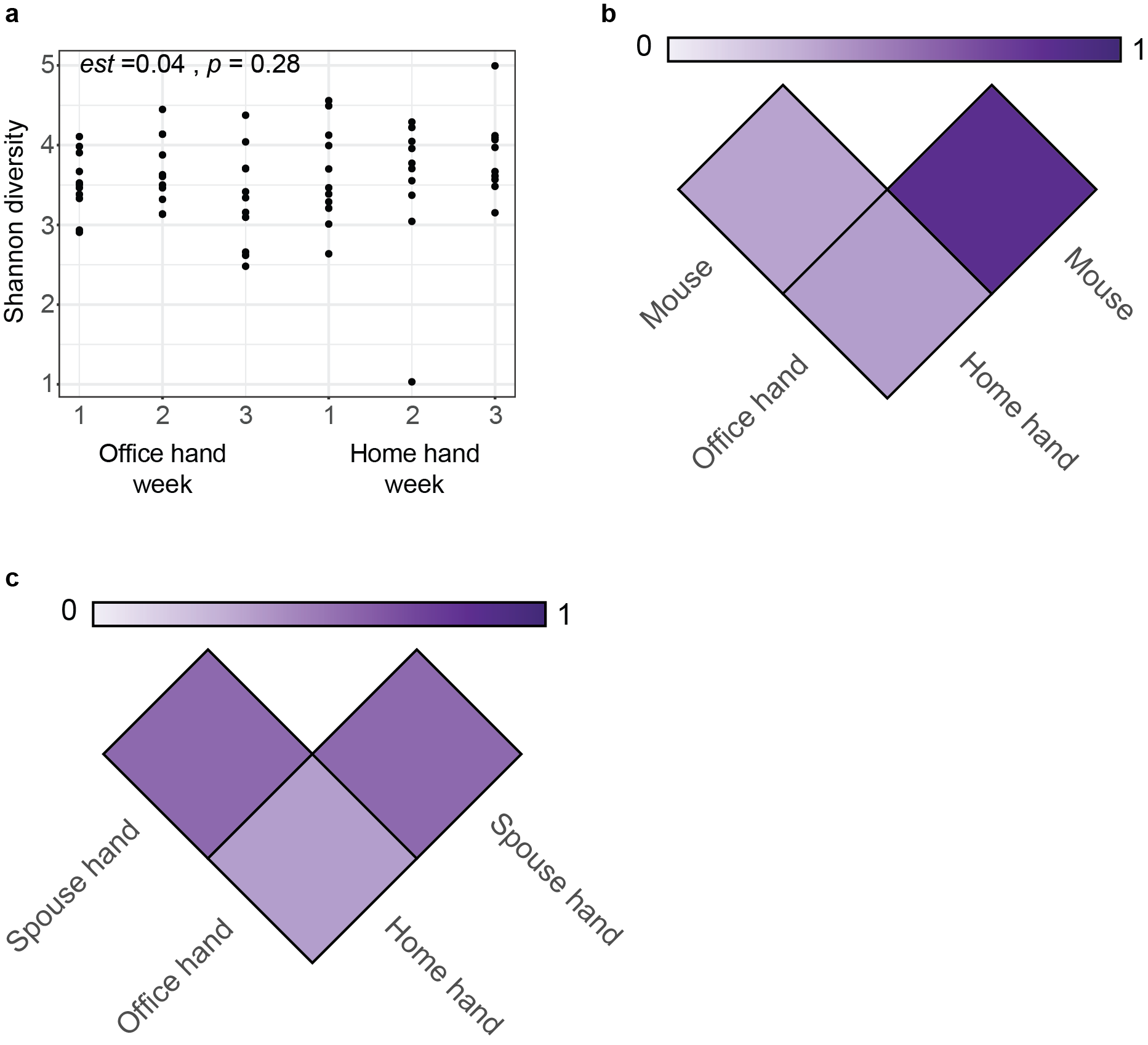


**Supplementary Figure 5. Cross-environment examination of longitudinal stability and microbial sharing across the office and home environments: a.** scatter plot of Shannon diversity within each week for participants that provided both office hand and home hand samples (*N* = 11) (*estimate* and *p* values from LMM). Sample sizes were, for the office environment for all weeks, (*n* = 11) and, for the home environment for week 1, (*n* = 11) and weeks 2 and 3 (*n* = 10); **b.** Heat map representing shared microbial communities from participants that provided hand samples from both office and home environments (*n* = 11)*.* Darker shades of purple represent higher proportions of microbial sharing. Samples sizes: mouse (*n* = 32); office hand (*n* = 33); home hand (*n* = 31); **c.** Heat map representing shared microbial communities from participants that provided hand samples from both the office and home environments and also provided partner samples (*n* = 6). Darker shades of purple represent a higher proportion of shared microbial communities. Sample sizes: office hand (*n* = 18); home hand (*n* = 18); partner hand (*n* = 17).


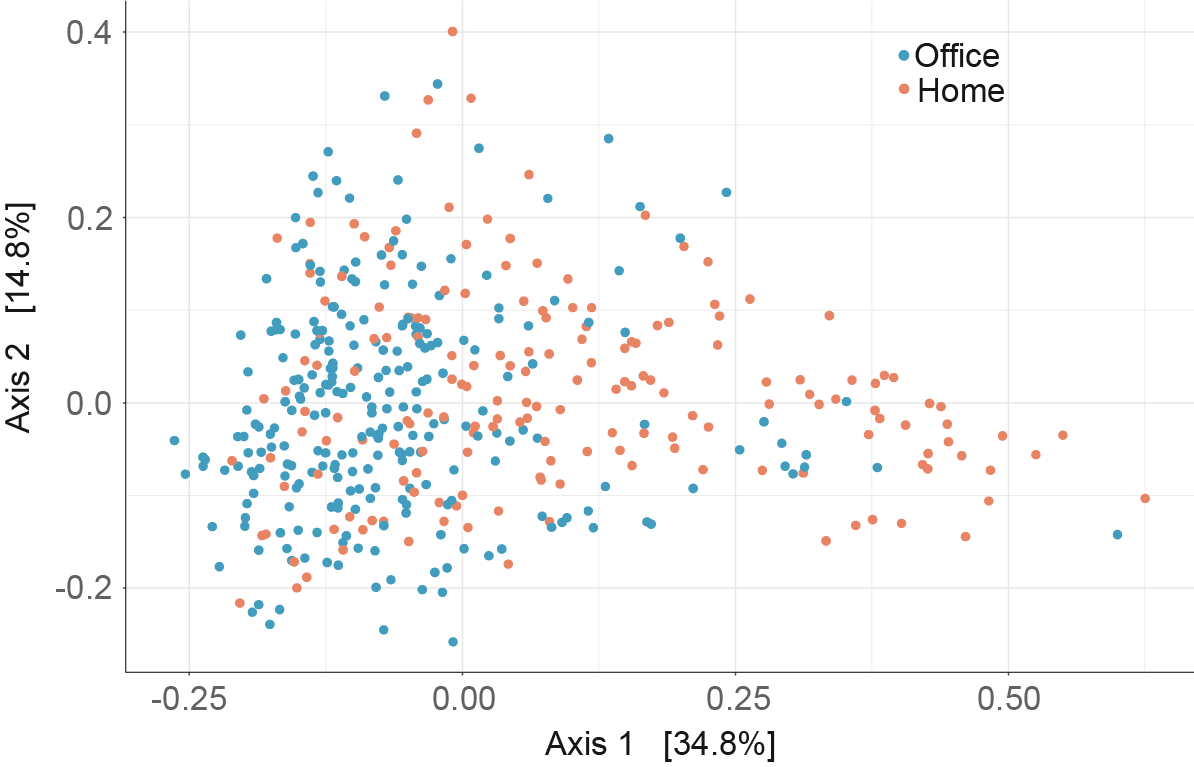


**Supplementary Figure 6.** **Beta diversity.** Principal coordinate analysis of weighted UniFrac dissimilarity in community composition among all samples.

## Supplementary Table 1. Sample sizes separated by sample type in the office environment

| Participant | Participant Hand | Mouse | Keyboard | Desk | Total |
| --- | --- | --- | --- | --- | --- |
| 1 | 3 | 3 | 3 | 3 | 12 |
| 2 | 3 | 2 | 2 | 2 | 9 |
| 3 | 3 | 3 | 3 | 3 | 12 |
| 4 | 3 | 3 | 2 | 3 | 11 |
| 5 | 3 | 3 | 3 | 3 | 12 |
| 6 | 2 | 3 | 3 | 2 | 10 |
| 7 | 3 | 3 | 3 | 3 | 12 |
| 8 | 3 | 3 | 3 | 3 | 12 |
| 9 | 3 | 3 | 3 | 3 | 12 |
| 10 | 3 | 3 | 3 | 3 | 12 |
| 11 | 3 | 3 | 3 | 3 | 12 |
| 12 | 3 | 3 | 3 | 3 | 12 |
| 13 | 3 | 3 | 3 | 3 | 12 |
| 14 | 3 | 3 | 3 | 3 | 12 |
| 15 | 3 | 3 | 3 | 2 | 11 |
| 16 | 3 | 3 | 3 | 3 | 12 |
| 17 | 3 | 3 | 3 | 3 | 12 |
| 18 | 3 | 3 | 3 | 3 | 12 |
| 19 | 2 | 3 | 3 | 3 | 11 |
| 20 | 3 | 3 | 3 | 3 | 12 |
| 21 | 3 | 3 | 3 | 3 | 12 |
| 22 | 3 | 3 | 3 | 3 | 12 |
| Total | 64 | 65 | 64 | 63 | 256 |

## Supplementary Table 2. Sample sizes separated by sample type in the home environment

| Participant | Participant  hand | Spouse hand | Nightstand | Bathroom | Bedroom  floor | Living room  floor | Total |
| --- | --- | --- | --- | --- | --- | --- | --- |
| 1 | 3 | 0 | 3 | 3 | 3 | 3 | 15 |
| 2 | 3 | 0 | 2 | 3 | 3 | 3 | 14 |
| 3 | 3 | 2 | 3 | 3 | 3 | 3 | 17 |
| 4 | 3 | 0 | 3 | 3 | 3 | 3 | 15 |
| 7 | 3 | 3 | 3 | 3 | 3 | 3 | 18 |
| 10 | 2 | 0 | 2 | 3 | 3 | 3 | 13 |
| 12 | 3 | 0 | 3 | 3 | 3 | 3 | 15 |
| 14 | 3 | 3 | 3 | 3 | 1 | 1 | 14 |
| 17 | 3 | 3 | 3 | 3 | 3 | 3 | 18 |
| 18 | 2 | 3 | 3 | 3 | 3 | 3 | 17 |
| 20 | 3 | 3 | 3 | 3 | 3 | 3 | 18 |
| Total | 31 | 17 | 31 | 33 | 31 | 31 | 174 |

## Supplementary Table 3. Sample sizes for Figure 3 represented as (training/test)

| Participant | Participant Hand | Mouse | Keyboard | Desk |
| --- | --- | --- | --- | --- |
| 1 | 64/9 | 65/9 | 64/9 | 63/9 |
| 2 | 64/6 | 65/7 | 64/7 | 63/7 |
| 3 | 64/9 | 65/9 | 64/9 | 63/9 |
| 4 | 64/8 | 65/8 | 64/9 | 63/8 |
| 5 | 64/9 | 65/9 | 64/9 | 63/9 |
| 6 | 64/8 | 65/7 | 64/7 | 63/8 |
| 7 | 64/9 | 65/9 | 64/9 | 63/9 |
| 8 | 64/9 | 65/9 | 64/9 | 63/9 |
| 9 | 64/9 | 65/9 | 64/9 | 63/9 |
| 10 | 64/9 | 65/9 | 64/9 | 63/9 |
| 11 | 64/9 | 65/9 | 64/9 | 63/9 |
| 12 | 64/9 | 65/9 | 64/9 | 63/9 |
| 13 | 64/9 | 65/9 | 64/9 | 63/9 |
| 14 | 64/9 | 65/9 | 64/9 | 63/9 |
| 15 | 64/8 | 65/8 | 64/8 | 63/9 |
| 16 | 64/9 | 65/9 | 64/9 | 63/9 |
| 17 | 64/9 | 65/9 | 64/9 | 63/9 |
| 18 | 64/9 | 65/9 | 64/9 | 63/9 |
| 19 | 64/9 | 65/8 | 64/8 | 63/8 |
| 20 | 64/9 | 65/9 | 64/9 | 63/9 |
| 21 | 64/9 | 65/9 | 64/9 | 63/9 |
| 22 | 64/9 | 65/9 | 64/9 | 63/9 |

## Supplementary Table 4. Sample sizes for Figure 4 represented as (training/test)

| Participant | Participant hand | Nightstand | Bathroom | Bedroom Floor | Living room floor |
| --- | --- | --- | --- | --- | --- |
| 1 | 31/12 | 31/12 | 33/12 | 31/12 | 31/12 |
| 2 | 31/11 | 31/12 | 33/11 | 31/11 | 31/11 |
| 3 | 31/12 | 31/12 | 33/12 | 31/12 | 31/12 |
| 4 | 31/12 | 31/12 | 33/12 | 31/12 | 31/12 |
| 7 | 31/12 | 31/12 | 33/12 | 31/12 | 31/12 |
| 10 | 31/11 | 31/11 | 33/10 | 31/10 | 31/10 |
| 12 | 31/12 | 31/12 | 33/12 | 31/12 | 31/12 |
| 14 | 31/8 | 31/8 | 33/8 | 31/10 | 31/10 |
| 17 | 31/12 | 31/12 | 33/12 | 31/12 | 31/12 |
| 18 | 31/12 | 31/11 | 33/11 | 31/11 | 31/11 |
| 20 | 31/12 | 31/12 | 33/12 | 31/12 | 31/12 |

# Data Availability Statement

The datasets for this study can be found in the QIITA under study ID 14787 https://qiita.ucsd.edu/study/description/14787 and EBI-ENA under the accession number PRJEB56766.
